# A Systematic Review and Meta-Analysis Finds Increased Blood Levels of All Forms of Ghrelin in Both Restricting and Binge-Eating/Purging Subtypes of Anorexia Nervosa

**DOI:** 10.3390/nu13020709

**Published:** 2021-02-23

**Authors:** Maria Seidel, Signe Markmann Jensen, Darren Healy, Aakriti Dureja, Hunna J. Watson, Birgitte Holst, Cynthia M. Bulik, Jan Magnus Sjögren

**Affiliations:** 1 Department of Medical Epidemiology and Biostatistics, Karolinska Institutet, Stockholm, 171 65 Solna, Sweden; Maria.seidel@uniklinikum-dresden.de (M.S.); cynthia_bulik@med.unc.edu (C.M.B.); 2 Division of Psychological and Social Medicine and Developmental Neurosciences, Faculty of Medicine, Technische Universität Dresden, 1099 Dresden, Germany; 3 Research Unit Eating Disorders, Psychiatric Center Ballerup, Maglevænget 32, 2750 Ballerup, Denmark; signe_m_ipsen@hotmail.com (S.M.J.); mkl314@alumni.ku.dk (D.H.); aakriti.dureja.01@regionh.dkc (A.D.); 4 Department of Psychiatry, University of North Carolina at Chapel Hill, Chapel Hill, NC 27599, USA; hunna_watson@med.unc.edu; 5 School of Psychology, Curtin University, Perth U1987, Australia; 6 Division of Paediatrics, University of Western Australia, Perth 6907, Australia; 7 Department of Biomedical Sciences, University of Copenhagen, 1353 Copenhagen, Denmark; holst@sund.ku.dk; 8 Department of Nutrition, University of North Carolina at Chapel Hill, Chapel Hill, NC 27599, USA; 9 Department of Clinical Medicine, University of Copenhagen, 2200 N Copenhagen, Denmark

**Keywords:** anorexia nervosa, ghrelin, desacyl ghrelin, acyl ghrelin, meta-analysis, systematic review, eating disorders

## Abstract

Anorexia nervosa (AN) is a severe psychiatric condition associated with high mortality and chronicity. The hunt for state, trait, subtyping, and prognostic biomarkers is ongoing and the orexigenic hormone ghrelin and its different forms, acyl ghrelin and desacyl ghrelin, have been proposed to be increased in AN, especially in the restrictive subtype. A systematic literature search was performed using established databases up to 30 November 2020. Forty-nine studies met inclusion criteria for cross-sectional and longitudinal meta-analyses on total ghrelin, acyl ghrelin, and desacyl ghrelin. All forms of ghrelin were increased in the acute stage of anorexia nervosa during fasting compared to healthy controls. Previous notions on differences in ghrelin levels between AN subtypes were not supported by current data. In addition, a significant decrease in total ghrelin was observed pre-treatment to follow-up. However, total ghrelin levels at follow-up were still marginally elevated compared to healthy controls, whereas for acyl ghrelin, no overall effect of treatment was observed. Due to heterogeneity in follow-up designs and only few data on long-term recovered patients, longitudinal results should be interpreted with caution. While the first steps towards a biomarker in acute AN have been completed, the value of ghrelin as a potential indicator of treatment success or recovery status or its use in subtype differentiation are yet to be established.

## 1. Introduction

Anorexia nervosa (AN) is a severe psychiatric disorder that typically begins during adolescence and is characterized by extreme weight loss or failure to gain weight appropriately for age, due to relentless control of food intake, intense fear of weight gain, and a distorted body image [1]. Individuals with acute AN exhibit multiple endocrine abnormalities that are commonly associated with starvation, including elevated stress hormones, hypogonadotropic hypogonadism, and adipocytokine disturbances [2,3]. Current knowledge suggests that many of the observed abnormalities normalize with weight restoration [4]. One of the endocrine hormones that is affected in AN is ghrelin, the only known peripherally produced and centrally active orexigenic hormone involved in gut–brain signalling for appetite control and energy balance [5]. Ghrelin is an endogenous ligand, secreted from oxyntic glands in the gastric fundus [5], that acts via the growth hormone secretagogues receptor 1a (GHSR1a) stimulating release of growth hormone (GH) from the pituitary, which in turn releases insulin-like growth factor 1 (IGF-1) as a response [6]. Of relevance to AN is that GHSR1a is expressed in the arcuate nucleus, in neurons that also express agouti-related peptide (AgRP) and neuropeptide Y (NPY), both of which are involved in regulating food intake [7]. The acyl form of ghrelin, being orexigenic, activates GHSR1a, which stimulates food intake [8], reduces insulin secretion leading to hyperglycaemia [9], and stimulates gastric motility [10]. The desacyl form of ghrelin has been described as anorexigenic and may counterbalance acyl ghrelin [11,12], probably acting as an agonist at the supraphysiological levels [13,14]. However, desacyl ghrelin has also been described as an inactive form of ghrelin, i.e., not associated with any biological activity [13]. For the last two decades, plasma levels of total ghrelin have been repeatedly found to be increased in acute AN [15]. Comparing AN patients with body-mass index (BMI)-matched, constitutionally lean people, one study showed significantly elevated plasma ghrelin levels in those with AN [16] supporting a potential role of ghrelin beyond the compensation of the undernourished state in this patient population. Furthermore, ghrelin levels have been found to positively correlate with the amount of physical activity in humans [17], which also have been observed in the activity-based animal model of anorexia (ABA) [18,19]. Interestingly, there have been studies finding higher fasting mean plasma levels of total ghrelin in individuals with binge-eating/purging behaviour [20] suggesting a potential influence of the number of binge/purge cycles as well as loss-of-control eating on the plasma concentration [21,22]. Furthermore, acyl ghrelin and desacyl ghrelin have been found to be increased in AN in some studies [23,24]. However, findings are heterogeneous, with AN subtypes, BMI differences, and methodological heterogeneity across studies possibly influencing the results [25].

During weight restoration in AN findings have been more heterogeneous, as ghrelin levels were reported to significantly decrease in several [16,26,27] but not all studies [28], and the size of the reduction might even differ between AN subtypes [29]. Also, correlations with BMI were not consistently reported across studies [26,29]. However, follow-up time-points were included with varying changes in BMI as well as duration of interventions and refeeding, thus results might not be directly comparable. Further, the question of normalization after therapeutic interventions still remains unanswered. 

As outlined, several studies have reported on ghrelin levels in AN; however, few attempts have been made to systematically pool and analyse these data to obtain a clearer estimate of the pattern of alterations both in the acute phase and during treatment. Ghrelin may have the potential to serve as a state, and possibly as a trait biomarker that could also be of considerable clinical relevance as an index of recovery. Accordingly, we aimed to systematically review the literature to investigate the quality of evidence and the levels of the various forms of ghrelin present in plasma, in acute AN as well as during, and after treatment, compared to healthy controls (HC).

## 2. Materials and Methods

The protocol was written and registered with the International Prospective Register of Systematic Reviews (PROSPERO): CRD42018100206. We used the Preferred Reporting Items for Systematic Reviews and Meta-Analyses guidelines (PRISMA [30]).

### 2.1. Eligibility Criteria

Studies were independently examined for eligibility by several authors (A.D., D.H., J.M.S., M.S., and S.J.) according to PICOs criteria [31]. The population (P) of interest was adolescents or adults with AN, and total ghrelin, acyl ghrelin, and desacyl ghrelin were outcomes (O). We included studies published before 30 November 2020, written in English. The following studies were eligible: original publications, case series, randomized controlled trials, cross-sectional studies, and cohort studies. Investigations into acute responses of ghrelin to standardized meals or glucose solutions were not considered in the scope of this review.

### 2.2. Search Strategy

The latest literature search was conducted on 30 November 2020 and was based on material/literature available in the electronic databases PubMed, EMBASE, and PsycINFO. The search terms included “Anorexia nervosa” OR “Anorexia” OR “Caloric restriction” OR “Appetite disorder(s)” AND “Ghrelin” OR “Acyl ghrelin” OR “Desacyl ghrelin” OR “GHRL-protein” OR “Ghrelin precursor” OR “Motilin related peptide precursor” OR “MLTRP”. In addition, the reference lists of all included publications were screened to identify additional eligible publications. For further information on the search terms, see Supplementary Materials 1.1.

### 2.3. Study Selection

**Figure 1:**
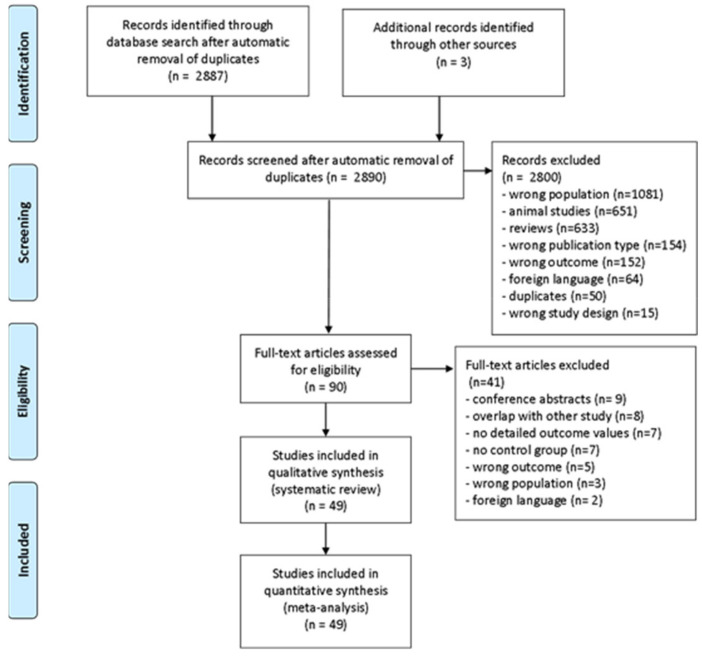
Preferred Reporting Items for Systematic Reviews and Meta-Analyses (PRISMA) flow diagram of study screening and inclusion.

Study selection is summarized in the PRISMA flow diagram (Figure 1). References were imported into EndNote X9. Following removal of duplicates, the remaining references were imported into Rayyan QCRI [32]. Using Rayyan QCRI’s “blind mode”, titles and abstracts were screened independently by at least two authors (A.D., D.H., J.M.S., M.S., or S.J.). Reviewers rated whether studies met the inclusion criteria by: (a) reviewing titles and abstracts and (b) conducting a full-text review. At least two reviewers evaluated each publication. The selection of individual papers was done according to the selection criteria in Supplementary Materials, Table S1. In the case of multiple or overlapping publications, we selected the one with the newest date or the largest dataset. The size of the dataset was prioritized over newer publication date, in case of conflict. Any disagreement was resolved through consensus discussion with the senior scientist (J.M.S.).

### 2.4. Data Extraction and Synthesis

Following study selection, available data (i.e., author name, publication year, AN and HC sample size, study design (cross-sectional, intervention, randomized control trial), diagnostic criteria (DSM-IV (including DSM-IV-TR); DSM-5; ICD-10 [1,33,34,35]); AN subtype (restricting, binge-eating/purging); AN and HC age; AN and HC BMI; ghrelin estimation method; unit of ghrelin; and plasma levels of total ghrelin, acyl ghrelin, and desacyl ghrelin) were extracted from the included papers using a data extraction template (see Supplementary Materials, Table S2), and were cross-checked by two reviewers (M.S. and J.M.S.). Some publications omitted raw ghrelin values (e.g., by presenting results in a figure); therefore, attempts were made to contact the corresponding authors for these data. If data were not provided or authors were not reachable, these studies were excluded from the meta-analysis. Missing values on descriptive data did not lead to exclusion of studies.

### 2.5. Risk of Bias in Individual Studies

To accommodate the types of bias inherent in different study designs, two scales were utilized; the Cochrane scale [36] was used to evaluate the risk of bias in randomized control trials (RCTs). The assessment of each type of bias is categorized into three groups (low, unclear, high) based on four main domains: selection bias, performance bias, detection bias, and reporting bias (see Supplementary Materials Table S3). For observational studies, the Newcastle-Ottawa Scale (NOS; [37]) for cross-sectional studies was used. The NOS consists of nine items grouped into three sections relating to the quality of an observational study (Supplementary Materials Table S4). For each outcome of interest, validity scores were evaluated as follows: Low quality = ≤5; Medium quality = 6–7; High quality = 8–9. To rate the levels of evidence, the “level of evidence tool” (as of March 2009) from the Oxford Centre for Evidence-based Medicine (www.cebm.ox.ac.uk/resources/levels-of-evidence) (accessed on 15 January 2021) was used. Level of evidence assessment was based on the methodological quality of the design, validity and applicability to patient care and ranges from 1 (highest level of evidence, e.g. systematic review or meta-analysis of RCTs) to 5 (lowest level of evidence, e.g. expert opinion; see Supplementary Materials Table S5). This process was completed independently by four reviewers (S.J., D.H. A.D., J.M.S).

### 2.6. Statistical Analysis

Meta-analyses and meta-regressions were conducted using the “metafor” and “robumeta” packages for the open-source software R (RStudio, version 1.3.959). For cross-sectional comparisons between acute AN patients (pre-treatment/baseline) and HCs, inverse variance-weighted random-effects meta-analyses were used. Standardized mean differences (SMD; Hedges’ adjusted g) with 95% confidence interval (CI) were used as effect sizes, since different units of ghrelin measures were reported in the primary studies. Between-study heterogeneity (τ^2^) was calculated using the method-of-moments estimator provided in Hedges et al. [38]. For studies reporting separate values for AN subtypes but only one HC value, we re-used the respective HC for both subtypes. Henceforth, to account for the dependence of sample sizes (e.g., multiple effect sizes per study), we conducted random-effects meta-analysis using robust variance estimation (RVE) [38,39]; using the “robumeta” package in R and included the recommended adjustment for small sample sizes [40]. RVE is based on weighted correlation estimation. The method is distribution free and provides valid point estimates, standard errors, and hypothesis tests even when the degree and structure of dependence between effect sizes is unknown [38,41]. Further sensitivity analyses were conducted to ensure the robustness of the results assuming varying correlation coefficients (rho).

For the longitudinal analyses, two different analytic approaches were applied. The first approach included all studies with pre-treatment values (T1) and one or more follow-up time points during intervention or post-treatment (T2) for AN patients. For these studies, we calculated a standardized mean change between pre-treatment and follow-up outcomes. The second approach included all studies providing follow-up data in the AN sample as well as reports on HC samples. We calculated an SMD for all available time-points. To account for dependence of sample sizes in the longitudinal analyses, we conducted random-effects meta-analyses using RVE and adjustment for small sample sizes and conducted sensitivity analyses to verify the robustness of the results at different rho values. Follow-up data were eligible for inclusion in longitudinal analyses if they occurred two or more weeks after the original measurement.

To examine the data further, we conducted meta-regression with the moderators of mean age, assay type (enzyme-linked immunosorbent assay, ELISA; radioimmunoassay, RIA; enzyme immunoassay, EIA; other assays (multiplex)), and quality of evidence to investigate whether these were related to calculated effect sizes. For the longitudinal models, we additionally conducted meta-regression adding mean age/age at baseline, assay type, BMI at baseline, BMI delta (BMI at baseline − BMI at follow-up), and duration between baseline and follow-up (in days) as moderators, first separately, then in one combined model.

## 3. Results

### 3.1. Study Search and Selection

The database search identified 2890 unique publications for abstract screening, which lead to the exclusion of 2800 papers (as illustrated in Figure 1). Following the review of full-texts, a final sample of 49 studies (1.7%) were identified for data extraction and meta-analysis (see Supplementary Materials Table S6 for exclusion details). For the studies included in the longitudinal assessment, only data following psychotherapeutic and (free) refeeding were included. For studies that provided medical interventions (such as ghrelin, oestrogen, or relamorelin administration) only placebo conditions were included if available. We were not informed of any additional published datasets after contacting study authors for additional or missing data.

### 3.2. Study Characteristics

Among the 49 studies (including a total of 1031 acute or recovered AN patients and 1002 HCs), 35 reported total ghrelin, 16 reported acyl ghrelin, and 11 reported desacyl ghrelin. Sample sizes ranged from 5 to 95 for the AN groups and from 6 to 115 for the HC groups. Patients with acute or recovered AN were on average 23.15 years (SD: 3.81, range: 15.9–32.6*), and HCs were 24.31 years (SD: 5.83, range: 14.8–48.5). All but two studies [42,43] were conducted on females only. The diagnosis of AN was made according to the DSM-IV (73.48%), DSM-5 (20.4%), or ICD-10 (2.04%) criteria, with two studies not indicating which diagnostic manual was used [26,44]. The blood samples were analysed via RIA (57.14%), ELISA (30.62%), EIA (8.16%), or other assays (2.04%; Supplementary Materials, Table S2). Twenty-five studies (51.03%) utilized a case–control or cross-sectional design (including multiple diagnostic groups), and 19 studies (38.78%) included a combination of case–control/cross-sectional components with longitudinal data at follow-up. Two studies (4.08%) reported follow-up data only, and three additional studies (6.12%) were randomized placebo–control designs. Of the 49 studies, 16 (32.64%) strictly included individuals with the restricting subtype of AN, one study (2.04%) only included individuals with the binge-eating/purging subtype, 11 studies (22.45%) included mixed samples, and the majority (n = 18; 36.74%) did not report which subtype of AN was studied, which probably indicated that subtypes were mixed.

With regard to quality of evidence, 18 studies (36.74%) were of low quality, 27 (55.11%) of medium quality, and one study (2.04%) of high quality according to the NOS. Three studies (6.12%) assessed with the Cochrane scale were of high quality (see Supplementary Materials, Tables S3, S4 and S6). Comprehensive summary data from eligible studies are presented in the data extraction table (Supplementary Materials, Table S7).

### 3.3. Meta-Analysis

#### 3.3.1. Fasting Total Ghrelin Levels in Acute AN

**Figure 2:**
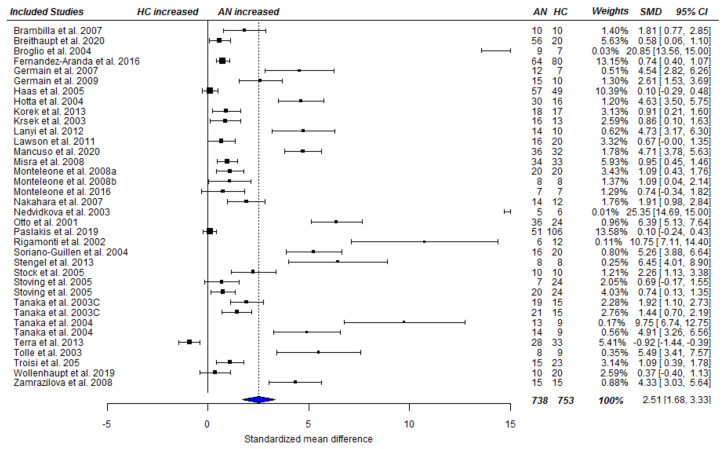
Forest plot of cross-sectional comparison of AN patients at baseline (pre-treatment; T1) and HCs on total ghrelin. AN = anorexia nervosa; HC = healthy control; SMD = standardized mean difference, CI = confidence interval. Studies listed more than once reported separate values for AN subtypes.

**Figure 3:**
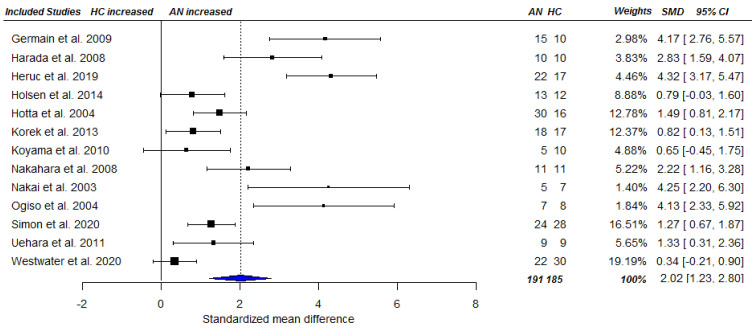
Forest plot of cross-sectional comparison of AN patients at baseline (pre-treatment; T1) and HCs on acyl ghrelin. AN = anorexia nervosa; HC = healthy control; SMD = standardized mean difference, CI = confidence interval.

**Figure 4:**
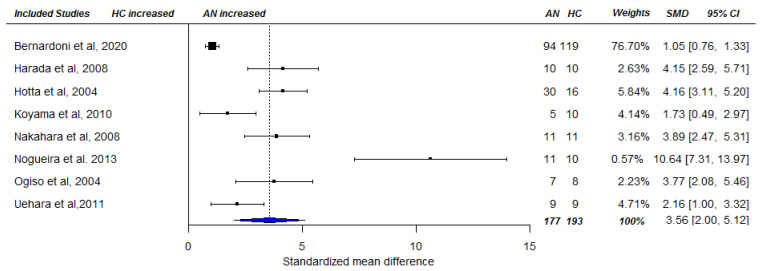
Forest plot of cross-sectional comparison of AN patients at baseline (pre-treatment; T1) and HCs on desacyl ghrelin. AN = anorexia nervosa; HC = healthy control; SMD = standardized mean difference, CI = confidence interval.

A cross-sectional random-effects meta-analysis was conducted including 33 studies [9,11,16,21,22,26,27,28,29,42,43,44,45,46,47,48,49,50,51,52,53,54,55,56,57,58,59,60,61,62,63,64,65], and 36 effect sizes that were based on 738 AN patients and 705 HCs, providing AN = 738 and HC = 753 data points for analysis. Applying RVE, the analysis revealed a statistically significantly higher total ghrelin level among acute AN patients compared with HC SMD_total_ = 2.51, 95% CI_total_ = [1.68, 3.33], *p* < 0.001), see Figure 2. Heterogeneity among studies was high (Ƭ^2^ = 2.35, I^2^ = 94.16%). The same pattern of results emerged for acyl ghrelin SMD_acyl_ = 2.02, CI_acyl_ = [1.14, 2.89], *p* < 0.001 (based on data of 13 studies [23,44,47,49,66,67,68,69,70,71,72,73,74] and effect sizes, including 191 AN patients, and 185 HC individuals as well as data points), see Figure 3. Again, heterogeneity of the pooled estimate was high (Ƭ^2^ = 1.26, I^2^ = 85.77%). Similarly, pooled effect sizes of eight studies ([49,66,69,70,71,74,75,76] based on 167 AN cases and 193 HC individuals and data points) reporting on desacyl ghrelin revealed a significantly higher level in acute AN patients compared with that in HCs (SMD_desacyl_ = 3.56, CI_desacyl_ = [1.67, 5.46], *p* = 0.003), see Figure 4. Heterogeneity of the pooled effect size was high (Ƭ^2^ = 3.54, I^2^ = 92.48%). The sensitivity analyses showed that τ^2^ and subsequently the pooled effect size estimates were relatively robust to different values of rho (Supplementary Materials, Table S8A–C).

Using meta-regression, adding mean age, assay type, and quality of evidence to the models (each first added separately, then combined at the same step) did not reveal a significant effect of any of the predictors used, see Supplementary Materials, Table S9A–C. Thirteen studies on total ghrelin used samples reporting values for strictly defined AN restricting (n = 10) or AN binge-eating/purging subtypes (n = 3) and contained a total of 193 acute AN patients and 192 HCs. A meta-regression including AN subtype as a moderator did not reveal a significant effect on effect sizes (coefficient subtype: −0.56 CI [−9.41, 8.20], *p* > 0.05).

#### 3.3.2. Fasting Ghrelin Levels in AN in Longitudinal Studies

For the longitudinal analyses, we first compared pre-treatment (baseline, T1) total ghrelin values to follow-up time-points (T2) among AN patients only, for descriptive data of included studies, see Table 1. Of the nine studies reporting total ghrelin at baseline and follow-up [16,26,28,29,48,58,70,77,78], four (44.44%) included patients admitted to an inpatient program at a clinic, including refeeding and psychotherapy [26,27,29,48]. Two studies (22.22%) included patients receiving outpatient psychotherapy and nutritional treatment [28,58] and three studies (33.33%) followed up on individuals with AN who did not receive treatment [16,77,78]. Inclusion criteria for follow-up was dependent on a specific weight gain [16,26,29,58], reaching a specific caloric intake [27] or passing a specific time-period after admission [28,48,77,78].

**Figure 5:**
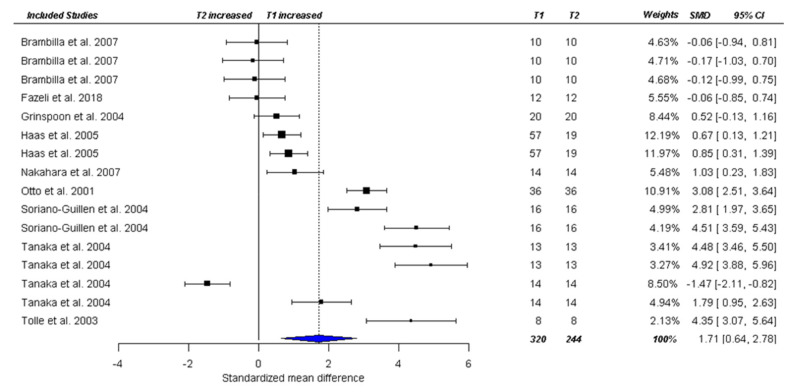
Forest plot of longitudinal comparison of acute AN patients at baseline (T1) and follow-up (T2) on total ghrelin. Studies shown more than once had more than one follow-up occasion. T1 = at baseline (pre-treatment); T2 = at follow-up. SMD = Standardized change score baseline—follow-up, CI = confidence interval.

Mean duration between baseline and follow-up was 94.62 days (for details see data extraction table: Supplementary Materials, Table S7). Four studies [28,29,48,58] reported multiple follow-ups. The nine studies provided 16 follow-up time points, which included a total of 200 patients at baseline and 162 at follow-up in the analysis (providing n = 320 T1 and n = 244 T2 data points). Between baseline and follow-up, total ghrelin decreased significantly (positive SMD values indicate a decrease in total ghrelin to the follow-up time point), SMD_total_=1.71, CI [0.45, 2.97], *p* = 0.014), see Figure 5. Heterogeneity of the pooled effect size was high (Ƭ^2^ = 2.70, I^2^ = 94.83%), and sensitivity analyses showed robustness of Ƭ^2^ in dependence of rho (Supplementary Materials, Table S8D). The results from a subsample of six studies providing 11 effect sizes (due to missing data) showed no association between moderators (age, BMI at baseline, BMI delta, duration to follow-up) and effect size (Table 2). A second meta-regression looking at assay type and quality of evidence, showed a significant effect for quality of evidence on effect sizes (SMD = −1.11, CI [−1.73–0.49], *p* = 0.001, in the quality only model. Results indicate that effect sizes between baseline and follow-up got larger (stronger decrease in ghrelin at T2) with reduced quality of study. Quality of study was no longer significant in the combined model (see Supplementary Materials, Table S9D).

**Figure 6:**
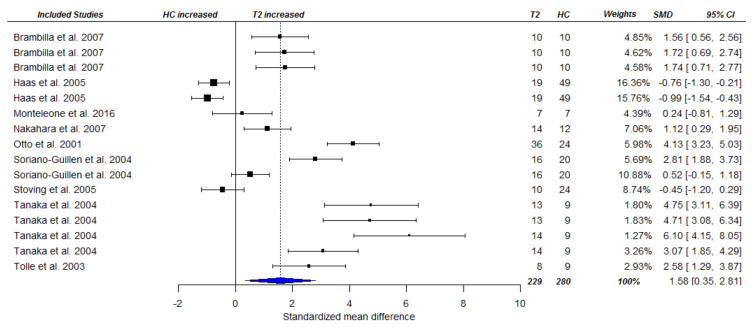
Forest plot of cross-sectional comparison between AN patients at follow-up (T2) and HCs on total ghrelin. Studies shown more than once had more than one follow-up occasion, or AN subtype.

The second approach compared the same data of AN patients during or after treatment (post-treatment or follow-up) to HCs, to establish whether ghrelin levels were still elevated in AN patients in comparison to HCs. Several studies provided multiple follow-up time-points [28,29,48,58]. All studies included in the longitudinal assessment above, except one that did not provide data on HCs [77], were included again. In addition to studies with follow-ups on acute AN patients as mentioned above, we also included two cross-sectional studies that compared weight-restored AN individuals with HCs [42,60]. The average follow-up duration was 93 days post-baseline. Pooling data resulted in the inclusion of nine studies yielding 16 effect sizes (based on 175 AN patients and 133 HCs, yielding 229 and 280 data points, respectively) for the meta-analysis. Compared to HCs, AN patients had significantly higher total ghrelin levels (SMD = 1.58, CI [0.13, 3.03], *p* = 0.036; see Figure 6). Heterogeneity was high (Ƭ^2^ = 3.47, I^2^ = 94.48%). Sensitivity analysis confirmed the robustness of the results under different assumptions for rho in both approaches (Supplementary Materials, Table S8E).

The two different approaches (change from baseline to follow-up and follow-up to HCs) were repeated for a longitudinal analysis of acyl ghrelin values. Longitudinal analysis of acyl ghrelin in five studies [67,69,74,78,79] provided data at seven follow-up time points, including a total of 77 AN patients (providing 135 data points). Four studies included patients in an inpatient setting, including a nutritional rehabilitation program (n = 2) and nutritional program combined with cognitive behavioural therapy (n = 2) and one study did not provide treatment. Average duration of follow-up was 44.14 days (and ranged between 14 and 90 days). Pooled data from the five studies showed no significant difference in acyl ghrelin level between pre-treatment and follow-up (SMD = −0.24, CI [−1.04, 0.56], *p* > 0.05). Heterogeneity was moderate (Ƭ^2^ = 0.29, I^2^ = 66.01%) and sensitivity analysis showed robustness of Ƭ^2^ (Supplementary Materials, Table S8F). Comparison of acyl ghrelin in AN patients at follow-up compared to HCs was possible in five studies [67,68,69,74,80] (yielding five effect sizes, including n = 57 AN patients and n = 63 HC individuals and data points) and revealed no significant difference between AN patients and HCs (SMD = 1.92, CI [−1.61, 5.45], *p* > 0.05) and high heterogeneity (Ƭ^2^ = 4.41, I^2^ = 93.81%) and robustness of Ƭ^2^ (Supplementary Materials, Table S8F). None of the meta-regression models revealed a significant moderator effect of age, assay type, quality of study, or duration of intervention on effect size (see Supplementary Materials, Table S9F,G). BMI and BMI delta could not be examined as moderators because of a lack of available data. There was not sufficient data on follow-up levels of desacyl ghrelin in AN patients to perform meta-analysis.

## 4. Discussion

### 4.1. Ghrelin at the Acute State

Despite numerous studies investigating fasting blood levels of ghrelin in AN [15,25,81], to the best of our knowledge, this is the first meta-analysis of all forms of ghrelin in AN that also addresses longitudinal changes. The results confirmed elevated fasting blood levels in acute AN compared to that in HCs for total ghrelin, and the acyl and desacyl forms of ghrelin. While elevated ghrelin levels have been suggested to be an adaptive response to hunger and cachectic states [82,83], prolonged increases in blood levels of ghrelin have been described as associated with ghrelin resistance [68], i.e., failure to respond with appropriate behaviour to appetite stimulating signals, or even ghrelin infusion [84]. However, since the levels of total ghrelin have been found to be decreased in BMI-matched lean individuals compared to that in AN patients [16,44,46], it may be that there are other factors beyond a low BMI and undernourished state that underlie elevated ghrelin in AN. Differences between these populations could be related to behavioural as well as physiologic aspects that contrast constitutionally lean individuals from AN, with specific reference to their different food intake and psychological profiles. For example, lean individuals do not exhibit clinical features such as persistent efforts to reduce energy intake, amenorrhea, distorted body image, or fear of weight gain. Moreover, lean individuals display an equilibrated energy metabolism similar to that of healthy controls, which might result in normalized ghrelin levels [44,46]. Earlier theories have also suggested that increased levels of total ghrelin may especially be due to an increase in inactive, i.e., desacyl ghrelin (altered active vs. inactive ratio) [49,85], which may counteract the metabolic effects of the active acyl ghrelin [9]. However, results of the current meta-analysis also showed significant increases in acyl ghrelin during the acute state, possibly opposing this theory. Moreover, one study showed desacyl ghrelin to be particularly associated with BMI and physical activity [79].

Another reason for altered ghrelin levels in AN could be genetic factors as suggested by Dardennes et al. [86], who studied 114 trios (individuals with AN and both of their parents). Transmission disequilibrium was observed for the Leu72Met single-nucleotide polymorphism (SNP) of the preproghrelin gene, and the polymorphisms were preferentially transmitted in trios in the binge-eating/purging subtype of AN [87]. In the general population, Met72GHREL carriers display the lowest BMI and the lowest fat mass; with less visceral fat and a lower fasting respiratory quotient, indicating a greater utilization of fat as an energy substrate [88]. Further studies using genome-wide methodology are required to explore genetic contributions to dysregulated ghrelin levels in AN.

Our understanding of how ghrelin regulates biological functioning and shapes behaviour is in its infancy since the activities of the GHSR1a receptor are complex [89], which is consistent with its widespread distribution in the brain [90,91]. Although the role of ghrelin in the regulation of energy homeostasis and food intake is widely acknowledged, more recent studies have focused on ghrelin as a potential modulator of cognitive functioning such as reward-based decision-making [56,75], which has been shown to be altered in this patient population [92]. The role of ghrelin in reward processing is thought to occur via its strong connections to the mesolimbic dopamine system [93]. For example, ghrelin injection was associated with increased dopamine in the nucleus accumbens [94] as well as heightened activation in key regions associated with pleasure and reward [95]. It is via this pathway that ghrelin is able to increase the incentive value of food [96] but also other reward-motivated behaviours such as alcohol consumption and drug use [15,97]. In patients with AN, high levels of ghrelin have been associated with better cognitive performance on the Iowa gambling task (reward sensitivity) [56] or with decreased delay discounting in AN (patients with high ghrelin more often chose the delayed reward) [75]. On the other hand, others have associated high ghrelin levels not only with increased impulsivity (in rodents [98]) but also with increased reward sensitivity [99,100] or reduced activity in areas associated with regulatory control (dorsolateral prefrontal cortex, [101]) in other populations. These mixed findings highlight the complexity of ghrelin signalling and its numerous interactions. Generally, those alterations in self-regulatory decision-making may point towards a link between the underlying biology and observed behaviours of individuals, which warrants further research and might even offer new avenues for the development of innovative treatment strategies.

The results of the meta-regression did not reveal an effect of AN subtype on differences in ghrelin between AN patients and HCs. As most studies reported mixed samples of the restricting and the binge-eating/purge subtype or failed to report on AN subtype, only few studies could be identified that compared ghrelin levels to HCs by AN subtype. One study found that total ghrelin was increased in the binge-eating/purging subtype compared to that in the restricting subtype [21], whereas other studies failed to replicate this finding reporting similarly increased levels of total ghrelin in both subtypes [60,73]. Since AN subtypes have not been a focus in the included studies on ghrelin, adequate evidence does not yet exist to determine whether the subtypes of AN differ in their ghrelin level, as well as in ghrelin normalization after treatment.

### 4.2. Ghrelin at Follow-Up

In the longitudinal analysis, total ghrelin levels in AN patients showed a significant decrease during treatment; however, levels were still significantly elevated when comparing the follow-up time points to HCs. Studies included in the meta-analysis varied in the duration between baseline and follow-up (ranging from 14 days to 12 months) and the treatments delivered. In addition, the heterogeneity as a result of differences in caloric intake during renourishment, the combination with cognitive-behaviour-oriented therapy (CBT), and inpatient/outpatient settings as well as free refeeding, might have introduced considerable variance across studies, which might have been only accounted for in parts by the meta-regression. Therefore, results of the longitudinal assessment should be interpreted with caution, warranting further research, possibly using more frequent sampling during follow-up in order to better synchronize and interpret results across studies.

Furthermore, longitudinal findings regarding the active form, acyl ghrelin, were inconclusive, possibly due to a lack of statistical power and small sample sizes or higher instability of acyl ghrelin. The results showed neither a change in acyl ghrelin from T1 to T2 nor alterations between T2 and HC data, which contradicts results from the cross-sectional analysis showing increased acyl ghrelin in acute AN. However, the duration of follow-up was also considerably lower (on average 44 days for acyl ghrelin vs. 94 days for total ghrelin), suggesting that recovery was only in the early stages. Studies on the changes of the desacyl from during or after treatment did not provide adequate data for a meta-analysis. Of note, while most data included in the longitudinal meta-analyses were obtained during or shortly after treatment, data on ghrelin levels in long-term (>6 months) weight recovered individuals were scarce. One study focused on total ghrelin [60], two on acyl ghrelin [68,80], and one on desacyl ghrelin [75]. While only one study did not show any differences between long-term recovered AN patients and HCs [60], the other still showed increased ghrelin levels even after long-term recovery [68,75], with the longest duration of recovery being 28 years [80]. It is not yet possible to conclude whether ghrelin constitutes a state marker only present during the undernourished and acute state of the illness or whether alterations in ghrelin levels might be present on a trait level.

Although our understanding of the (neuro)biological mechanisms of AN has increased over the past years, relapse rates and hospital readmission are still high, many patients never fully recover [102,103,104,105] and we are still lacking effective evidence-based treatment approaches. To overcome this challenge, clinicians need better tools than subjective assessment of symptoms to determine stage of illness, remission, and recovery. Biomarker tools can provide objective indices of the physiological state through accurate and reproducible measurements. They can also reflect objective and often quantifiable characteristics of the complex biological processes involved in the disease or a specific phenotype of interest, which enable better diagnosis in light of symptom overlap (e.g., AN to constitutional leanness, or between subtypes) as well as improve the accuracy of prognosis. Further, illness progression is thought to be linked to changes in underlying pathophysiology, which could be better quantified by biomarkers. There has been growing interest in applying a staging model for AN [106] to represent different stages of the disorder from acutely ill to fully recovered. However, while experts reached, for example, consensus on a five-stage model in AN, there is still little agreement regarding appropriate biomarkers (besides BMI) that could be used to differentiate these stages of illness. Our meta-analysis suggests that ghrelin might be a reasonable candidate to consider in future research.

The following limitations should be considered when evaluating this meta-analysis: First, most of the included studies were cross-sectional and only afforded an examination of baseline ghrelin levels. Additional studies that take repeated measures from baseline (pre-treatment) through weight restoration, psychological recovery, and follow-up are necessary to fully explicate the pattern of ghrelin dysregulation and the time course of changes in AN, and to determine the extent to which ghrelin is a state and/or trait marker. While ghrelin is not yet part of the standard diagnostic procedure for AN, adding it might enable a more detailed investigation of short- vs. long-term changes during therapeutic intervention. Further, so far, a careful mapping of important covariates, such as caloric intake and nutrient composition or changes in self-reported appetite and body-fat composition, has been missing but would provide useful knowledge about the appetite-related processes in AN. This further extends to acute responses of ghrelin levels to nutritional intake [81] or recent advances into treatments with ghrelin or ghrelin agonists, which have showed mixed results [78,84,107,108] and could be explored further.

## 5. Conclusions

This systematic review and meta-analysis found evidence for elevated fasting blood levels of all forms of ghrelin (total, acyl, desacyl) during the acute state of AN compared to that in HCs, supporting evidence of increased ghrelin values as a state biomarker in AN. No effect of AN subtype on the differences between AN patients and HCs in fasting blood levels of ghrelin was found, although only few studies have investigated this aspect. Furthermore, levels of total ghrelin significantly decreased during follow-up, whereas acyl ghrelin did not show this effect, possibly also due to the limited power and sample sizes of included studies. However, comparing longitudinal data from patients with AN to HCs still revealed marginally increased total ghrelin levels in AN. As the included studies were heterogeneous concerning design and inclusion criteria for follow-up and the data of long-term recovered individuals are lacking, data should be interpreted with caution.

While the first steps towards evaluating ghrelin as a biomarker have been completed, the value of ghrelin as a potential indicator of treatment success or recovery status, its use in subtype differentiation, or as basis for prognosis is yet to be established.

## Figures and Tables

**Table 1:** Mean, standard deviation, and minimum and maximum of characteristics of participants included in the longitudinal assessment of total ghrelin (n = 200). BMI = body-mass index, BMI delta = BMI at baseline − BMI at follow-up, duration to follow-up is given in days.

	Mean	SD	Min	Max
Age at baseline	22.56	3.72	17.00	28.9
BMI at baseline	14.94	1.49	12.40	17.8
BMI delta	−1.71	1.24	−0.1	−4.40
Duration to follow-up	94.62	89.56	30	365

**Table 2:** Meta-regression for longitudinal changes in total ghrelin from baseline (T1) to follow-up (T2). In this model, assay type was not included since all the studies included (except one) used radioimmunoassays (RIAs). BMI delta = BMI baseline − BMI follow-up. Duration was transformed to and entered as days. SE = standard error; CI, L = confidence interval, lower bound; CI, U = confidence interval, upper bound.

	Estimate	SE	*p*	95% CI, L	95%, CI, U
Age	0.19	0.37	0.64	−1.13	1.52
BMI baseline	−1.13	1.36	0.47	−5.78	3.51
BMI delta	0.83	1.41	0.60	−3.86	5.53
Duration to follow-up	−0.01	0.01	0.74	−0.07	0.06

## Data Availability

No new data were created or analyzed in this study. Data sharing is not applicable to this article.

## Supplementary Materials

The following are available online at https://www.mdpi.com/2072-6643/13/2/709/s1, Supplementary Materials 1.1. Tables S1–S9.
