# Supplementary material for: A Systematic Review and Meta-Analysis Finds Increased Blood Levels of All Forms of Ghrelin in Both Restricting and Binge-Eating/Purging Subtypes of Anorexia Nervosa
Source: Nutrients. 2021 Feb 23;13(2):709. doi: 10.3390/nu13020709 (PMC7926807; doi:10.3390/nu13020709)
Supplement: Supplementary file 1 [file nutrients-13-00709-s001.pdf]

Supplementary material Seidel et al.

1.1. Search terms

PUBMED:
((("Anorexia"[Mesh] OR "Anorexia Nervosa"[Mesh] OR "Bulimia"[Mesh] OR "Bulimia Nervosa"[Mesh] OR "Binge-Eating Disorder"[Mesh] OR "Caloric Restriction"[Mesh]) OR ("eating disorder" OR "eating disorders" OR Anorexia OR " Anorexia nervosa" OR "Binge-eating disorder" OR "binge eating disorder" OR "Binge-eating disorders" OR "binge eating disorders" OR bulimia OR "Bulimia nervosa" OR "caloric restriction" OR "appetite disorder" OR "appetite disorders" OR "feeding disorder" OR "feeding disorders")) AND ((Ghrelin OR ″GHRL Protein″ OR Ppghrelin OR ″Ghrelin-Obestatin Preprohormone″ OR ″Ghrelin Obestatin Preprohormone″ OR ″Motilin-Related Peptide Precursor″ OR ″Motilin Related Peptide Precursor″ OR PpMTLRP OR ″Ghrelin Precursor″ OR Obestatin OR ″Appetite-Regulating Hormone″ OR ″Appetite Regulating Hormone″ OR ″Motilin-Related Peptide″ OR ″Motilin Related Peptide″ OR ″Gastric MLTRP″ OR acylghrelin OR ″acyl Ghrelin″ OR des-acylghrelin OR ″des-acyl ghrelin″) OR ("Ghrelin"[Mesh])))

Psycinfo
(Eating disorder OR eating disorders OR anorexia OR anorexia nervosa OR binge-eating disorder OR binge-eating disorders OR bulimia OR bulimia nervosa OR caloric restriction OR appetite disorder OR appetite disorders OR feeding disorder OR feeding disorders) AND (Ghrelin OR acylghrelin OR des-acylghrelin OR GHRL protein OR ppghrelin OR Ghrelin-obsestatin preprohormone OR Motilin-related petide precursor OR PpMTLRP OR Ghrelin Precursor)

Embase
1. ("eating disorder" or "eating disorders" or Anorexia or " Anorexia nervosa" or "Binge-eating disorder" or "binge eating disorder" or "Binge-eating disorders" or "binge eating disorders" or bulimia or "Bulimia nervosa" or "caloric restriction" or "appetite disorder" or "appetite disorders" or "feeding disorder" or "feeding disorders").mp. [mp=title, abstract, heading word, drug trade name, original title, device manufacturer, drug manufacturer, device trade name, keyword, floating subheading word, candidate term word]
2. exp anorexia/
3. exp anorexia nervosa/
4. exp eating disorder/
5. exp binge eating disorder/
6. exp bulimia/
7. exp caloric restriction/
8. exp appetite disorder/
9. exp feeding disorder/
10. 1 or 2 or 3 or 4 or 5 or 6 or 7 or 8 or 9
11. (Ghrelin or acylghrelin or des-acylghrelin or GHRL protein or ghrelin-obsestain preprohormone or motilin-related peptide precursor or PpMTLRP or ghrelin precursor).mp. [mp=title, abstract, heading word, drug trade name, original title, device manufacturer, drug manufacturer, device trade name, keyword, floating subheading word, candidate term word]
12. exp ghrelin/
13. 11 or 12
14. 10 and 13

Table S1: In an exclusion criteria for selection of studies

Inclusion criteria	Exclusion criteria
Anorexia nervosa	Review
Ghrelin	No available ghrelin data
Acyl ghrelin	Case report
Desacyl ghrelin	No access through the University of Copenhagen
Adults or adolescents	Non-English papers
	Animal research
	In vitro

Table S2: Data extraction template

Data extracted
Author name
Year
Sample size AN and HC
Design type
Diagnostic criteria
Intervention type
AN subtype
Ghrelin total
Ghrelin acyl
Ghrelin desacyl
Ghrelin unit
Assay type
Age: AN and HC
BMI: AN and HC

Table S3: Risk of bias assessment through Cochrane Collaboration:

Cochrane Collaborations tool for assessing risk of bias	Selection bias Random sequence generation	Selection bias Allocation concealment	Performance bias Blinding of participants and personnel	Detection bias Blinding of outcome assessment	Attrition bias Incomplete outcome data	Reporting bias Selective reporting	Other bias	Total bias assessment:
**Brambilla et al, 2007**	Unclear risk	Low risk	Low risk	Low risk	Low risk	Low risk	-	Low risk = high quality
**Grinspoon et al, 2004**	Low risk	Low risk	Low risk	Unclear risk	Low risk	Low risk	-	Low risk = High quality
**Fazeli et al, 2018**	Low risk	Unclear risk	Low risk	Unclear risk	Unclear risk	Low risk		Low risk = High quality

Table S4: Quality assessment using Newcastle-Ottawa Quality Assesment Scale (NOS) (cross-sectional studies)

	Selection				Comparability	Outcome		
**Newcastle-Ottawa Quality Assesment Scale (Cross-sectional studies)**	**1. Representativeness of the cases**	**2. Sample size**	**3. Ascertainment of exposure**	**4. Non-response rate**	**5. Comparability of cases and controls**	**6. Assessment of outcome**	**7. Follow-up long enough for outcome to occur**	**Total score: Quality of study**
**Bernadoni et al, 2020**	A (Non-random sampling) *	A (justified) *	A (validated) *	A (comparability satisfactory) *	A (Age) *	A (independent blind assessment) *	A (Statistical test appropriate) *	7
**Breithaupt et al, 2020**	A (Non-random sampling)*	A (justified) *	A (validated) *	A (comparability satisfactory) *	None	A (independent blind assessment) *	A (Statistical test appropriate) *	6
**Broglio et al, 2004**	A (non-random sampling) *	B (not justified)	A (validated) *	A (comparability satisfactory) *	None	A (independent blind assessment) *	A (Statistical test appropriate) *	5
**Duriez et al, 2020**	A (non-random sampling)*	A (justified) *	A (validated) *	A (comparability satisfactory) *	None	A (independent blind assessment) *	A (Statistical test appropriate) *	6
**Fernandez-Arande et al., 2016**	A (non-random sampling)*	A (justified) *	A (validated) *	A (comparability satisfactory) *	A (Age) *	A (independent blind assessment) *	A (Statistical test appropriate) *	7
**Germain et al, 2007**	A (Non-random sampling)*	B (not justified)	A (validated) *	A (comparability satisfactory) *	A (Age) *	A (independent blind assessment) *	A (Statistical test appropriate) *	6
**Germain et al, 2009**	A (Non-random sampling)*	B (not justified)	A (validated) *	A (comparability satisfactory) *	A (Age) *	A (independent blind assessment) *	A (Statistical test appropriate) *	6
**Haas et al, 2005**	A (Non-random sampling)*	A (justified) *	A (validated) *	A (comparability satisfactory) *	A (Age) * A (Sex) *	A (independent blind assessment) *	A (Statistical test appropriate) *	8
**Harada et al, 2008**	B (Non-random sampling)	B (not justified)	A (validated) *	A (comparability satisfactory) *	None	A (independent blind assessment) *	A (Statistical test appropriate) *	4
**Heruc et al, 2019**	A (Non-random sampling)*	B (not justified)	A (validated) *	A (comparability satisfactory) *	A (Age) *	A (independent blind assessment) *	A (Statistical test appropriate) *	6
**Holsen et al, 2014**	A (Non-random sampling)*	B (not justified)	A (validated) *	A (comparability satisfactory) *	None	A (independent blind assessment) *	A (Statistical test appropriate) *	5
**Hotta et al, 2004.**	B (non-random sampling)	A (justified) *	A (validated) *	A (comparability satisfactory) *	A (Age) *	A (independent blind assessment) *	A (Statistical test appropriate) *	6
**Korek et al, 2013**	B (non-random sampling)	B (not justified)	A (validated) *	A (comparability satisfactory) *	None	A (independent blind assessment) *	A (Statistical test appropriate) *	4
**Koyama et al, 2010**	A (Non-random sampling)*	B (not justified)	A (validated) *	A (comparability satisfactory) *	A (Age) *	A (independent blind assessment) *	A (Statistical test appropriate) *	6

**Krsek et al, 2003**	A (Non-random sampling)*	B (not justified)	A (validated) *	A (comparability satisfactory) *	None	A (independent blind assessment) *	A (Statistical test appropriate) *	5
**Lanyi et al, 2012**	A (Non-random sampling)*	B (not justified)	A (validated) *	A (comparability satisfactory) *	None	A (independent blind assessment) *	A (Statistical test appropriate) *	5
**Lawson et al, 2011**	A (Non-random sampling)*	B (not justified)	A (validated) *	A (comparability satisfactory) *	None	A (independent blind assessment) *	A (Statistical test appropriate) *	5
**Manusco et al, 2020**	A (Non-random sampling)*	A (justified) *	A (validated) *	A (comparability satisfactory) *	None	A (independent blind assessment) *	A (Statistical test appropriate) *	6
**Misra et al, 2008**	A (Non-random sampling) *	A (justified) *	A (validated) *	A (comparability satisfactory) *	A (Age) *	A (independent blind assessment) *	A (Statistical test appropriate) *	7
**Monteleone et al, 2008a**	A (Non-random sampling) *	B (not justified)	A (validated) *	A (comparability satisfactory) *	None	A (independent blind assessment) *	A (Statistical test appropriate) *	5
**Monteleone et al, 2008b**	A (Non-random sampling) *	B (not justified)	A (validated) *	A (comparability satisfactory) *	A (Age) *	A (independent blind assessment) *	A (Statistical test appropriate) *	6
**Monteleaone et al, 2016**	A (Non-random sampling) *	B (not justified)	A (validated) *	A (comparability satisfactory) *	None	A (independent blind assessment) *	A (Statistical test appropriate) *	5
**Nakahara et al, 2007**	A (Non-random sampling) *	B (not justified)	A (validated) *	A (comparability satisfactory) *	A (Age) *	A (independent blind assessment) *	A (Statistical test appropriate) *	6
**Nakahara et al, 2008**	A (Non-random sampling) *	B (not justified)	A (validated) *	A (comparability satisfactory) *	A (Age) *	A (independent blind assessment) *	A (Statistical test appropriate) *	6
**Nakai et al, 2003**	A (Non-random sampling) *	B (not justified)	A (validated) *	A (comparability satisfactory) *	A (Age) *	A (independent blind assessment) *	A (Statistical test appropriate) *	6
**Nedvidkova et al, 2003**	A (Non-random sampling) *	B (not justified)	A (validated) *	A (comparability satisfactory) *	None	A (independent blind assessment) *	A (Statistical test appropriate) *	5
**Nogueira et al, 2013**	A (Non-random sampling) *	B (not justified)	A (validated) *	A (comparability satisfactory) *	None	A (independent blind assessment) *	A (Statistical test appropriate) *	5
**Ogiso et al, 2011**	A (Non-random sampling) *	B (not justified)	A (validated) *	A (comparability satisfactory) *	A (Age) *	A (independent blind assessment) *	A (Statistical test appropriate) *	6
**Otto et al, 2001**	A (Non-random sampling) *	A (justified) *	A (validated) *	A (comparability satisfactory) *	A (Age) *	A (independent blind assessment) *	A (Statistical test appropriate) *	7
**Paslakis et al, 2019**	A (Non-random sampling) *	A (justified) *	A (validated) *	A (comparability satisfactory) *	None	A (independent blind assessment) *	A (Statistical test appropriate) *	6
**Rigamonti et al, 2002**	A (Non-random sampling) *	B (not justified)	A (validated) *	A (comparability satisfactory) *	None	A (independent blind assessment) *	A (Statistical test not appropriate)	4
**Simon et al., 2020**	A (Non-random sampling) *	A (justified) *	A (Yes) *	A (comparability satisfactory) *	A (Age) *	A (independent blind assessment) *	A (Statistical test appropriate) *	7

**Soriano-Guille, 2004**	A (Non-random sampling) *	B (not justified)	A (validated) *	A (comparability satisfactory) *	None	A (independent blind assessment) *	A (Statistical test appropriate) *	5
**Stengel et al., 2013**	A (Non-random sampling) *	B (not justified)	A (validated) *	A (comparability satisfactory) *	BMI	A (independent blind assessment) *	A (Statistical test appropriate) *	5
**Stock et al, 2005**	A (Non-random sampling) *	B (not justified)	A (validated) *	A (comparability satisfactory) *	None	A (independent blind assessment) *	A (Statistical test appropriate) *	5
**Stojilkkovic-Drobnjak et al. 2019**	A (Non-random sampling) *	B (not justified)	A (validated) *	A (comparability satisfactory) *	A (BMI) *	A (independent blind assessment) *	A (Statistical test appropriate) *	6
**Stoving et al, 2007**	A (Non-random sampling) *	A (justified) *	A (validated) *	A (comparability satisfactory) *	A (Age) *	A (independent blind assessment) *	A (Statistical test appropriate) *	7
**Tanaka et al, 2003c**	A (Non-random sampling) *	B (not justified)	A (validated) *	A (comparability satisfactory) *	A (Sex) *	A (independent blind assessment) *	A (Statistical test appropriate) *	6
**Tanaka et al, 2004**	A (Non-random sampling) *	B (not justified)	A (validated) *	A (comparability satisfactory) *	A (Age) *	A (independent blind assessment) *	A (Statistical test appropriate) *	6
**Terashi et al, 2011**	A (Non-random sampling) *	B (not justified)	A (validated) *	A (comparability satisfactory) *	None	A (independent blind assessment) *	A (Statistical test appropriate) *	5
**Terra et al, 2013**	A (Non-random sampling)*	B (not justified)	A (validated) *	A (comparability satisfactory) *	A (Age) *	A (independent blind assessment) *	A (Statistical test appropriate) *	6
**Tolle et al, 2003**	A (Non-random sampling)*	B (not justified)	A (validated) *	A (comparability satisfactory) *	A (Age) *	A (independent blind assessment) *	A (Statistical test appropriate) *	6
**Troisi et al, 2005**	A (Non-random sampling)*	B (not justified)	A (validated) *	A (comparability satisfactory) *	A (Age) * A (BMI) *	A (independent blind assessment) *	A (Statistical test appropriate) *	7
**Westwater et al, 2020**	A (Non-random sampling)*	B (not justified)	A (validated) *	A (comparability satisfactory) *	None	A (independent blind assessment) *	A (Statistical test appropriate) *	5
**Wollenhaupt et al, 2019**	A (Non-random sampling)*	B (not justified)	A (validated) *	A (comparability satisfactory) *	None	A (independent blind assessment) *	A (Statistical test appropriate) *	5
**Uehara et al, 2011**	A (Non-random sampling)*	B (not justified)	A (validated) *	A (comparability satisfactory) *	A (Age) *	A (independent blind assessment) *	A (Statistical test appropriate) *	6
**Zamrazilova et al, 2008**	A (Non-random sampling) *	B (not justified)	A (validated) *	A (comparability satisfactory) *	A (Age) *	A (independent blind assessment) *	A (Statistical test appropriate) *	6

* The asterisk is given when the item score meets a predefined criteria/level. The sum is given in the last column. The higher the score, the higher the quality of the study.

Table S5: Overview of evidence level for included studies

Study	Tool	Evidence Level (Oxford CEMB)	Study design
**Bernardoni et al., 2020**	NOS	Level 3B	Cross-sectional study
**Brambilla et al., 2007**	Cochrane collaboration	Level 1b	Randomized, double-blind placebo controlled design
**Breithaupt et al., 2020**	NOS	Level 3B	Cross-sectional study
**Broglio et al., 2004**	NOS	Level 3B	Cross-sectional study
**Duriez et al., 2020**	NOS	Level 2b	Cohort study
**Fazeli et al., 2018**	Cochrane collaboration	Level 2b	Randomized controlled study
**Fernandez-Aranda et al., 2016**	NOS	Level 3B	Cross-sectional study
**Germain et al., 2007**	NOS	Level 3B	Cross-sectional study
**Germain et al., 2009**	NOS	Level 3B	Cross-sectional study
**Grinspoon et al., 2004**	Cochrane collaboration	Level 1B	Randomized controlled study
**Haas et al., 2005**	NOS	Level 3B	Cross-sectional study
**Heruc et al., 2019**	NOS	Level 3B	Cross-sectional study
**Harada et al., 2008**	NOS	Level 3B	Cross-sectional study
**Holsen et al., 2014**	NOS	Level 3B	Cross-sectional study
**Hotta et al., 2004**	NOS	Level 3B	Cross-sectional study
**Korek et al., 2013**	NOS	Level 3B	Cross-sectional study
**Koyama et al., 2010**	NOS	Level 3B	Cross-sectional study
**Krsek et al., 2003**	NOS	Level 3B	Cross-sectional study
**Lanyi et al., 2012**	NOS	Level 3B	Cross-sectional study
**Lawson et al., 2011**	NOS	Level 3B	Cross-sectional study
**Mancuso et al., 2020**	NOS	Level 3B	Cross-sectional study
**Misra et a., 2008**	NOS	Level 3B	Cross-sectional study
**Monteleone et al., 2008a**	NOS	Level 3B	Cross-sectional study
**Monteleone et al., 2008b**	NOS	Level 3B	Cross-sectional study
**Monteleone et al., 2016**	NOS	Level 3B	Cross-sectional study
**Nakahara et al., 2007**	NOS	Level 3B	Cross-sectional study
**Nakahara et al., 2008**	NOS	Level 3B	Cross-sectional study
**Nakai et al., 2003**	NOS	Level 3B	Cross-sectional study
**Nedvidkova et al., 2003**	NOS	Level 3B	Cross-sectional study
**Nogueira et al., 2013**	NOS	Level 3B	Cross-sectional study
**Ogiso et al., 2011**	NOS	Level 3B	Cross-sectional study
**Otto et al., 2001**	NOS	Level 3B	Cross-sectional study
**Paslakis et al., 2019**	NOS	Level 3B	Cross-sectional study
**Rigamonti et al., 2002**	NOS	Level 3B	Cross-sectional study
**Simon et al., 2020**	NOS	Level 3B	Cross-sectional study
**Soriano-Guille., 2003**	NOS	Level 3B	Cross-sectional study
**Stengel et al., 2013**	NOS	Level 3B	Cross-sectional study
**Stock et al., 2005**	NOS	Level 3B	Cross-sectional study
**Stojilkkovic-Drobnjak et al., 2019**	NOS	Level §B	Cross-sectional study
**Stoving et al., 2007**	NOS	Level 3B	Cross-sectional study
**Tanaka et al., 2003c**	NOS	Level 3B	Cross-sectional study
**Tanaka et al., 2004**	NOS	Level 3B	Cross-sectional study
**Terra et al., 2013**	NOS	Level 3B	Cross-sectional study
**Tolle et al., 2003**	NOS	Level 3B	Cross-sectional study
**Troisi et al., 2005**	NOS	Level 3B	Cross-sectional study
**Uehara et al., 2011**	NOS	Level 3B	Cross-sectional study
**Westwater, 2020**	NOS	Level 3B	Cross-sectional study
**Wollenhaupt et al., 2019**	NOS	Level 3B	Cross-sectional study
**Zamrazilova et al., 2008**	NOS	Level 3B	Cross-sectional study

NOS= Newcastle-Ottawa Scale; Oxford CEMB=Oxford Centre for Evidenced Based Medicine

Table S6: Reasons for excluding specific papers

Study	Titel	Reason
Baker et al. 2019	Correlation between gene expression change and hormone change during refeeding in Anorexia nervosa	Conference abstract
Baker et al. 2020	Metabolic Predictors of Length of Stay on an Eating Disorder Inpatient Unit	Conference abstract
Baskeran et al. 2016	Leptin secretory dynamics and associated disordered eating psychopathology across the weight spectrum	Overlap with Lawson et al. 2011
Beranova et al 2009	Neuropeptide Y, ghrelin and leptin plasma levels in anorexia nervosa patients and their changes during six-week refeeding	Non-English
Briatore et al. 2006	Acute plasma glucose increase, but not early insulin response, regulates plasma ghrelin	Wrong population
Bulant etl al. 2020	Changes of BMI, steroid metabolome and psychopathology in patients with anorexia nervosa during hospitalization	Reported values not accessible
Galmiche et al. 2020	Plasma Peptide Concentrations and Peptide-Reactive Immunoglobulins in Patients with Eating Disorders at Inclusion in the French EDILS Cohort	No control group/ No follow-up
Germain et al 2010	Somatic and psychological factors related to the body mass index of patients with anorexia nervosa	Overlap Germain et al. 2009
Germain et al. 2016	Interleukin-7 plasma levels in human differentiate anorexia nervosa, constitutional thinness and healthy obesity	Wrong outcome
Goebel-Stengel et al. 2013	The ghrelin activating enzyme ghrelin-O-acyltransferase (GOAT) is present in human plasma and expressed dependent on body mass index	Overlap Stengel et al. 2013
Hofmann et al 2016	Plasma kisspeptin and ghrelin levels are independently correlated with physical activity in patients with anorexia nervosa	No control group
Holsen et al. 2012	Subcortical food motivation circuitry dysfunction associated with endogenous active ghrelin levels in women with anorexia nervosa	Conference abstract
Hotta et al. 2009	Ghrelin Increases Hunger and Food Intake in Patients with Restricting-type Anorexia Nervosa: A Pilot Study	No control group

Hotta et al. 2012	Therapeutic potential of ghrelin in restricting-type anorexia nervosa	Overlap with Hotta et al. 2004
Janas-Kosik et al. 2007	Total ghrelin plasma level in patients with the restrictive type of anorexia nervosa	Reported values not accessible
Janas-Kozik et al. 2006	The plasma levels of orexigenic peptides and human platelets phospholipase D activity in anorexia nervosa patients	Non-English
Janas-Kozik et al. 2011	Plasma levels of leptin and orexin A in the restrictive type of anorexia nervosa	Wrong outcome
Karczewska-Kupczewska et al.2010	Increased suppression of serum ghrelin concentration by hyperinsulinemia in women with anorexia nervosa	Reported values not accessible
Kawai et al. 2008	Somatic and psychological factors related to the body mass index of patients with anorexia nervos	No control group
Kawai et al., 2017	Ghrelin activation and neuropeptide Y elevation in response to medium chain triglyceride administration in anorexia nervosa patients	No control group, not clear which nutritional supplement condition is "normal food"
Lawson et al,. 2012	Subcortical food motivation circuitry dysfunction associated with endogenous active ghrelin levels in women with anorexia nervosa	Overlap Lawson et al. 2011
Lofrano-Prado et al., 2016	Non-traditional biomarkers of eating disorder symptoms among female college students	Wrong population
Miljic et al., 2006	Ghrelin has partial or no effect on appetite, growth hormone, prolactin, and cortisol release in patients with anorexia nervosa	No control group
Miljic et al., 2007	Glucose metabolism during ghrelin infusion in patients with anorexia nervosa	No control group
Misra et al. 2005a	Secretory dynamics of ghrelin in adolescent girls with anorexia nervosa and healthy adolescents	Wrong outcome
Misra et al., 2005b	Ghrelin and bone metabolism in adolescent girls with anorexia nervosa and healthy adolescents	Wrong outcome
Misra et al., 2005c	Secretory dynamics of leptin in adolescent girls with anorexia nervosa and healthy adolescents	Wrong outcome
Otto et al. 2005	Postprandial ghrelin release in anorectic patients before and after weight gain	Overlap Otto et al. 2001
Plessow et al., 2018	Postprandial changes in ghrelin levels are associated with subsequent hedonic food intake and activation in the food reward pathway in healthy controls but not females with anorexia nervosa	Conference abstract

Ramoz et al. 2017	Genetic and neuroendocrinological biomarkers in anorexia nervosa and relatives	Conference abstract
Ramoz et al. 2018	Neuropeptide concentrations and epigenetic profiles of patients who remitted from anorexia nervosa: Prognostic biomarkers?	Conference abstract
Ramoz et al., 2015	Hunger and satiety signals in anorexia nervosa: Neuroendocrinological and genetic analyses	Conference abstract
Schneider et al., 2008	Effects of age, malnutrition and refeeding on the expression and secretion of ghrelin	Wrong population
Sedlackova et al., 2011	Changes of plasma obestatin, ghrelin and NPY in anorexia and bulimia nervosa patients before and after a high-carbohydrate breakfast	Reported values not accessible
Sedlackova et al., 2012	Comparison of a high-carbohydrate and high-protein breakfast effect on plasma ghrelin, obestatin, NPY and PYY levels in women with anorexia and bulimia nervosa	Reported values not accessible
Shiiya et al., 2002	Plasma Ghrelin Levels in Lean and Obese Humans and the Effect of Glucose on Ghrelin Secretion	Reported values not accessible
Stachowicz et al., 2011	The roles of ghrelin and orexin A in patients with the binge–purging type of anorexia nervosa: preliminary study	Conference abstract
Tanaka et al. 2003b	Eating pattern and the effect of oral glucose on ghrelin and insulin secretion in patients with anorexia nervosa	Overlap Tanaka et al. 2003c
Tanaka et al., 2003c	Fasting plasma ghrelin levels in subtypes of anorexia nervosa	Overlap Tanaka et al. 2003c
Uehara et al., 2005	Plasma des-acyl and acyl ghrelin in patients with eating disorders	Reported values not accessible
Wu et al, 2020	Stress and Appetite Hormones in Predicting Binge Eating in Anorexia Nervosa: A Longitudinal Study	Conference abstract

List of reasons for exclusion of specific studies. In case of not accessible data attempts were made to contact contributing authors to provide data. 52% of attempts were successful. For others there was either no response or no valid email address available.

Tabel S7: Data Extraction Table

Study	Gender	N (AN)	N (HC)	Subtype	Age AN	Age HC	Diagnostic method	Method assay	Assay spec.	BMI AN	BMI HC	Design	Samples	Ghrelin Form	Unit Ghrelin	Duration T2	Quality Level*	Evidence level
Bernardoni et al., 2020, AN	F	94	119	AN-R	16.1	18.6	DSM-5	ELISA	BioVendor	14.60	20.9	CS	AN, REC, HC	deacyl	pg/ml	Baseline	7	3b
Brambilla et al., 2007, T1	F	10	10	N/A	23	N/A	DSM-IV	RIA	Phoenix	16.30	22.3	R, DB PC	AN T1, HC	total	pg/ml	Baseline	High	1b
Brambilla et al., 2007, T2	F	10	10	N/A	23	N/A	DSM-IV	RIA	Phoenix	16.67	22.3	R, DB PC	AN T2, HC	total	pg/ml	1 month	High	1b
Brambilla et al., 2007, T3	F	10	10	N/A	23	N/A	DSM-IV	RIA	Phoenix	17.2	22.3	R, DB PC	AN T3, HC	total	pg/ml	2 months	High	1b
Brambilla et al., 2007, T4	F	10	10	N/A	23	N/A	DSM-IV	RIA	Phoenix	17.5	22.3	R, DB PC	AN T4, HC	total	pg/ml	3 months	High	1b
Breithaupt et al., 2020	F	36	20	AN-R/AN-BP	19	18.4	DSM-5	ELISA	Millipore	N/A	N/A	CS	AN(AN +Atypical), HC	total	pg/ml	Baseline	6	3b
Broglio et al., 2004	F	9	7	AN-R	24.2	30.6	DSM-IV	RIA	Phoenix B	14.70	20.3	CS/I	AN, HC	total	ug/ml	Baseline	5	3b
Duriez et al., 2020, T1	F	29	N/A	AN-R (59%)/AN-BP (41%)	25.8	N/A	DSM-5	EIA	Bertin	14.60	N/A	I	AN T1	acyl, deacyl	pg/ml	Baseline	6	2b
Duriez et al. 2020, T2	F	28	N/A	AN-R (59%)/AN-BP (41%)		N/A	DSM-5	EIA	Bertin		N/A	I	AN T2		pg/ml	partial weight recovery, 50-70% target BMI	6	2b
Duriez et al., 2020, T3	F	13	N/A	AN-R (59%)/AN-BP (41%)	26.2	N/A	DSM-5	EIA	Bertin	N/A	N/A	I	AN T3	acyl, deacyl	pg/ml	partial weight recovery 90% target BMI	6	2b
Duriez et al., 2020, T4	F	13	N/A	AN-R (59%)/AN-BP (41%)	26	N/A	DSM-5	EIA	Bertin	N/A	N/A	I	AN T4	acyl, deacyl	pg/ml	successful weight recovery 1 month post discharge	6	2b
Fazeli et al. 2018, T1	F	12	N/A	N/A	28.9	N/A	DSM-5	ELISA	Millipore	17.80	N/A	R controlled	AN T1	total, acyl, deacyl	pg/ml	Baseline	High	2b
Fazeli et al. 2018, T2	F	12	N/A	N/A	28.9	N/A	DSM-5	ELISA	Millipore	17.90	N/A	R controlled	AN T2	total, acyl, deacyl	pg/ml	4 weeks after starting treatment	High	2b
Fernandes-Aranda et al. 2016	F	64	80	AN-R (66%)/ AN-BP (34%)	24	22.6	DSM-5	ELISA	Millipore	17.40	21.6	CS	AN, YHC, OHC, OB	total	N/A	Baseline	7	3b

Germain et al., 2007	F	12	7	N/A	20.7	23	DSM-IV	RIA	Phoenix B	15.20	20.4	CS	AN, THIN, HC	total	pmol/L	Baseline	6	3b
Germain et al., 2009	F	15	9	AN-R	20.4	23.1	DSM-IV	ELISA	in-house	14.80	20.5	CS/I	AN, THIN, HC	total, acyl	pg/ml	Baseline	6	3b
Grinspoon et al. 2004	F	68	20	N/A	25.4	N/A	DSM-IV	RIA	Phoenix B	15.90	N/A	R controlled	IFG-1, Estrogen, IGF-1+Estrogen, Placebo	total	pg/ml	Baseline	High	1b
Haas et al., 2005, T1	F	57	49	N/A	25	25	DSM-IV	RIA	In house	14.60	22.3	CS	AN T1	total	pmol/L	baseline	8	3b
Haas et al., 2005, T2	F	19	49	N/A	25	25	DSM-IV	RIA	In house	16.8	22.3	CS	AN T2	total	pmol/L	34 days after first assessment	8	3b
Haas et al., 2005, T3	F	19	49	N/A	25	25	DSM-IV	RIA	In house	17.7	22.3	CS	AN T3	total	pmol/L	84 days after first assessment	8	3b
Harada et al., 2008	F	10	10	AN-R	21.9	23.5	DSM-IV	ELISA	Mitsubishi	13.43	21.6	CS/I	AN, HC	acyl, deacyl	pg/ml	baseline	4	3b
Heruc et al., 2019, T1	F	22	17	N/A	15.9	16.5	DSM-5	multiplex assay	Milliplex	18.10	22.50	CS/I	AN T1, HC	acyl	pg/ml	baseline	6	3b
Heruc et al., 2019, T2	F	22	17	N/A	15.9	16.5	DSM-5	multiplex assay	Milliplex	N/A	N/A	CS/I	AN T2, HC	acyl	pg/ml	2 weeks	6	3b
Holsen et al., 2014, AN	F	13	12	AN-R	21.8	21.6	DSM-IV	RIA	LINCO	18.10	22.5	CS	AN, REC, HC	acyl	pg/ml	baseline	5	3b
Holsen et al., 2014, REC	F	9	12	N/A	23.2	21.6	DSM-IV	RIA	LINCO	22.20	22.5	CS	AN, REC, HC	acyl	pg/ml	at least 6 months	5	3b
Hotta et al., 2004	F	30	16	AN-R(70%)/AN-BP (30%)	24	25.6	DSM-IV	ICT-EIA (total ghrelin) + ELISA	LINCO	15.54	20.28	CS/I	AN, HC	total, acyl, deacyl	nmol/L	baseline	6	3b
Korek et al., 2013	F	18	17	N/A	20-35	19-25	N/A	RIA	Millipore	16.50	21.1	CS/I	AN, OB, HC	total, acyl	pg/ml	baseline	4	3b
Koyama et al., 2010, T1	F	5	10	AN-R	22.4	25.2	DSM-IV	ELISA	Mitsubishi	12.17	20.97	CS/I	AN T1, HC	acyl, deacyl	pg/ml	baseline	6	3b
Koyama et al., 2010, T2	F	5	10	AN-R	22.7	25.2	DSM-IV	ELISA	Mitsubishi	13.93	20.97	CS/I	AN T2, HC	acyl, deacyl	pg/ml	8 weeks	6	3b
Krsek et al. 2003	F	16	13	N/A	26.6	29.2	DSM-IV	RIA	Phoenix CA	15.20	20.7	CS	AN, HC, Short bowel syndrome	total	ng/L	baseline	5	3b
Lanyi et al., 2012	F	11	10	N/A	19.3	19.6	DSM-IV	RIA	LINCO	17.10	22.2	CS	AN, OB, HC	total	ng/L	baseline	5	3b

Lawson et al., 2011	F	16	20	N/A	25.9	27.3	DSM-IV	RIA	LINCO	18.2	22.3	CS	AN, HC, OB, NW	total	pg/ml	baseline	5	3b
Mancuso et al., 2019	F	36	32	AN-R(69%)/AN-BP (31%)	19.6	17.5	K-SADS-PL + EDE	ELISA	Millipore	17	21.3	CS/I	AN, HC	total	pg/ml	baseline	6	3b
Misra et al., 2008	F	34	33	N/A	15.9	15	DSM-IV	RIA	Phoenix B	16.60	22.3	CS	AN, HC	No	pg/ml	baseline	7	3b
Monteleone et al. 2016, AN	F/M	7	7	AN-R (71%)/AN-BP (29%)	24	25.2	DSM-IV	EIA	Phoenix B	15.90	22.07	CS	AN, HC	total	ng/ml	baseline	5	3b
Monteleone et al. 2016, REC	F	7	7	AN-R (71%)/ AN-BP (29%)	23.7	25.2	DSM-IV	EIA	Phoenix B	19.40	22.07	CS	AN-REC, HC	total	ng/ml	2-14 weeks normal BMI	5	3b
Monteleone et al., 2008a	F	20	20	AN-R(35%)/AN-BP (65%)	23.7	23.6	DSM-IV	RIA	Phoenix B	16.40	21.1	CS	AN, BN, HC	total	pg/ml	baseline	5	3b
Monteleone et al., 2008b	F	8	8	AN-R(25%)/AN-BP (75%)	23.7	22.1	DSM-IV	RIA	Phoenix B	16.40	21.3	CS	AN, HC	total	pg/ml	baseline	6	3b
Nakahara et al., 2007, T1	F	14	12	AN-R	24.6	25.7	DSM-IV	RIA	In-hous	12.40	22.3	CS/I	AN T1, HC	total	pmol/L	baseline	6	3b
Nakahara et al., 2007, T2	F	14	12	AN-R	24.6	25.7	DSM-IV	RIA	In-house	16.8	22.3	CS/I	AN T2, HC	total	pmol/L	86 (+/- 26 days) days after admission	6	3b
Nakahara et al., 2008	F	11	11	AN-R	27.7	24.6	DSM-IV	RIA	In-house	12.40	21.8	CS	AN, OB, HC	acyl, deacyl	fmol/ml	baseline	6	3b
Nakai et al., 2003	F	5	7	AN-R	N/A	age-matched	DSM-IV	RIA	In-house	13.90	20.4	CS/I	AN, HC	acyl	fmol/ml	baseline	6	3b
Nedvidkova et al., 2003, T1	F	5	6	N/A	24.3	23	DSM-IV	RIA	Phoenix B	15.20	21.6	CS/I	AN T1, HC	total	pg/ml	baseline	5	3b
Nogueira et al., 2013, T1	F	10	11	AN-R	21.9	24.6	DSM-IV	ELISA	BioVendor	13.10	22.3	CS/I	AN T1, HC	deacyl	pg/ml	baseline	5	3b
Ogiso et al. 2011	F	7	8	AN-R	19.3	19	DSM-IV	ELISA	Mitsubishi	13.02	21.57	CS	AN, HC	acyl, deacyl	fmol/ml	baseline	6	3b
Otto et al., 2001, T1	F	36	24	N/A	25	22.9	N/A	RIA	Phoenix B	15.20	21.9	CS/I	AN T1, HC	total	pg/ml	baseline	7	3b
Otto et al., 2001, T2	F	36	24	N/A	25	22.9	N/A	RIA	Phoenix B	17.4	21.9	CS/I	AN T2, HC	total	pg/ml	discharge 66+/- 25 days	7	3b
Paslakis et al., 2019	F	51	106	AN-R (66%)/AN-BP (33%)	27.4	27.2	DSM-5	ELISA	Millipore	17.44	21.7	CS	AN, HC	total	pg/ml	baseline	6	3b
Rigamonti et al., 2002	F	6	12	N/A	17-18	27-39	DSM-IV	RIA	Phoenix B	13.1	21.1	CS	AN, OB, HC	total	pg/ml	baseline	4	3b
Simon et al., 2020	F	24	28	AN-R (83%)/AN-BP (17%)	23.5	24.6	DSM-5	ELISA	Millipore	15.48	21.87	CS	AN, HC, OB	acyl	pg/ml	baseline	7	3b
Soriano-Guillen et al., 2004, T1	F	16	20	N/A	17	17.3	DSM-IV	RIA	Phoenix B	-2.20	0.3	CS/I	AN T1, OB, HC	total	pg/ml	baseline	5	3b

Soriano-Guillen et al., 2004, T2	F	16	20	N/A	17	17.3	DSM-IV	RIA	Phoenix B	-1	0.3	CS/I	AN T2, OB, HC	total	pg/ml	25% of BMI at admission	5	3b
Soriano-Guillen et al., 2004, T3	F	16	20	N/A	18	17.3	DSM-IV	RIA	Phoenix B	-0.58	0.3	CS/I	AN T3, OB, HC	total	pg/ml	1 year	5	3b
Stengel et al., 2013	AN (F); HC(F/M)	8	8	N/A	26.1	48.5	ICD-10	ELISA	Phoenix	12.6	22.6	CS	AN, HC, OB	total	ng/ml	baseline	5	3b
Stock et al., 2005	F	10	10	AN-R	16.5	14.8	DSM-IV	RIA	Phoenix B	16.30	20.2	CS	AN, HC, OB	total	pg/ml	baseline	5	3b
Stojilkkovic-Drobnjak et al., 2019	F	11	15	N/A	pre (40-60)		DSM-IV	ELISA	MSD	20.60	22.1	CS	REC, HC	acyl	pg/ml	28 years	6	3b
Stoving et al., 2007, BP	F	7	24	AN-BP	26	29.9	DSM-IV	RIA	In-house	15.00	23.6	CS	AN-R, AN-BP, REC, HC	total	ug/L	baseline	7	3b
Stoving et al., 2007, R	F	20	24	AN-R	27.6	29.9	DSM-IV	RIA	In-house	15.30	23.6	CS	AN-R, AN-BP, REC, HC	total	ug/L	baseline	7	3b
Stoving et al., 2007, REC	F	10	24	N/A	23.9	29.9	DSM-IV	RIA	In-house	20.10	23.6	CS	AN-R, AN-BP, REC, HC	total	ug/L	at least 6 months	7	3b
Tanaka et al., 2003c, BP	F	19	15	AN-BP	24.6	22.1	DSM-IV	RIA	In-house	13.80	21.4	CS/I	AN-BP, BN, HC	total	pmol/L	baseline	6	3b
Tanaka et al., 2003c, R	F	21	15	AN-R	21.8	22.1	DSM-IV	RIA	In-house	13.30	21.4	CS/I	AN-R, BN, HC	total	pmol/L	baseline	6	3b
Tanaka et al., 2004, BP T1	F	13	9	AN-BP	25	21.9	DSM-IV	RIA	In-house	14.50	21.5	CS/I	E-AN, AN-BP T1, HC	total	pmol/L	baseline	6	3b
Tanaka et al., 2004, BP T2	F	13	9	AN-BP	25	21.9	DSM-IV	RIA	In-house	15.30	21.5	CS/I	E-AN, AN-BP T2, HC	total	pmol/L	15-42 days	6	3b
Tanaka et al., 2004, BP T3	F	13	9	AN-BP	25	21.9	DSM-IV	RIA	In-house	16.20	21.5	CS/I	E-AN, AN-BP T3, HC	total	pmol/L	discharge	6	3b
Tanaka et al., 2004, R T1	F	14	9	AN-R	18.4	21.9	DSM-IV	RIA	In-house	13.10	21.5	CS/I	E-AN, AN-R T1, HC	total	pmol/L	baseline	6	3b
Tanaka et al., 2004, R T2	F	14	9	AN-R	18.4	21.9	DSM-IV	RIA	In-house	13.90	21.5	CS/I	E-AN, AN-R T2, HC	total	pmol/L	15-42 days	6	3b
Tanaka et al., 2004, R T3	F	14	9	AN-R	18.4	21.9	DSM-IV	RIA	In-house	15.10	21.5	CS/I	E-AN, AN-R T3, HC	total	pmol/L	discharge	6	3b
Terra et al., 2013	F	28	33	AN-R	32.6	27.4	DSM-IV	EIA	LINCO	16.80	21.8	CS/I	AN, HC	total	ng/ml	baseline	6	3b
Tolle et al., 2003, T1	F	9	10	AN-R	17.2	23.2	DSM-IV	RIA	Phoenix B	14.60	21.5	CS/I	AN T1, THIN, HC	total	ng/L	baseline	6	3b
Tolle et al., 2003, T2	F	9	10	AN-R	17.2	23.2	DSM-IV	RIA	Phoenix B	17.90	21.5	CS/I	AN T2, THIN, HC	total	ng/L	N/A	6	3b
Troisi et al., 2005	F	15	23	AN-R(91%)/A	26.7	25.6	DSM-IV	RIA	LINCO	15.97	21.24	CS	AN, BN,	total	pg/ml	baseline	7	3b

				N-BP (9%)									BED, HC					
Uehara et al., 2011, T1	F	9	9	AN-R	21.3	23.9	DSM-IV	ELISA	Mitsubishi	12.71	21.96	CS/I	AN T1, HC	acyl, desacyl	pg/ml	baseline	6	3b
Uehara et al., 2011, T2	F	9	9	AN-R	21.4	23.9	DSM-IV	ELISA	Mitsubishi	13.99	21.96	CS/I	AN T2, HC	acyl, desacyl	pg/ml	2-3 weeks	6	3b
Westwater at al., 2020	F	22	30	AN-BP	24.6	23.9	DSM-5	RIA	Millipore	16.40	21.9	CS	AN, BN, HC	acyl	pg/ml	baseline	5	3b
Wollenhaupt et al., 2019	F	10	10	N/A	29.5	34.9	DSM-5	ELISA	Millipore	16.37	21.8	CS	AN, BN, BED	total	pg/ml	baseline	5	3b
Zamrazilova et al., 2008	F	15	15	AN-R	23.7	34.7	DSM-IV	RIA	LINCO	14.90	21.6	CS	AN, OB, HC	total	ng/L	baseline	6	3b

Abbreviations: AN=Anorexia nervosa, AN-BP=Anorexia nervosa binge eating/purging subtype; AN-R=Anorexia nervosa restricting subtype; BED=Binge-eating disorder; BMI=Body Mass Index; BN=Bulimia nervosa; Bertin=Bertin Bioreagent, Montigny-le-Bretonneaux, France; CS=Cross-sectional; DB=Double-blind; DSM=Diagnostic and Statistical Manual of mental disorders; E-AN=Anorexia nervosa patient submitted to emergent hospitalization (not included in the meta-analysis); ELISA=Enzyme Linked Immuno Sorbent Assay; F=Female; HC=Healthy Controls; I=Intervention; L=Liter, LINCO= LINCO Research, St. Charles, MO, USA; M=male; Milliplex=Milliplex® MAP Human; Mitsubishi=Mitsubishi Kagaku Iatron, Tokyo, Japan; Millipore=Millipore, Billerica, MA, USA; ml=milliliter; MSD= MSD® Human Active Ghrelin Assay; Meso Scale Discovery, Gaithersburg, MD, USA; N=number of; N/A=Not applicable/not reported; ng=nanogram; NW= NW+hypothalamic amenorrhea; OB=Obesity; PC=Placebo controlled; pg=picogram; Phoenix Pharmaceuticals, Burlingame, CA, USA; Phoenix=Phoenix Pharmaceuticals, Mountain View, USA; Phoenix B=Phoenix Pharmaceuticals, Inc., Belmont, CA, USA; pre=premenopausal; R=Randomized; RIA=Radio Immuno Assay; T1=baseline measurement of AN patients at acute state (pretreatment); T2=1st follow up time point after baseline; T3=2nd follow-up time point after baseline; T4=3rd follow-up time point after baseline. * Quality level was assessed with the Newcastle-Ottawa Quality Assessment Scale (NOS) or bias assessment through Cochrane collaboration for randomized placebo controlled trials;

Table S8: Results of sensitivity analyses with varying rho

	Rho	.00	.20	.40	.60	.80	1.00
**A) Cross-sectional total ghrelin**	Intercept	2.51	2.51	2.51	2.51	2.51	2.51
	Std. Error	0.42	0.42	0.42	0.42	0.42	0.42
	Tau.sq	2.35	2.35	2.35	2.35	2.35	2.35
**B) Cross-sectional acyl ghrelin**	Intercept	2.02	2.02	2.02	2.02	2.02	2.02
	Std. Error	0.40	0.40	0.40	0.40	0.40	0.40
	Tau.sq	1.26	1.26	1.26	1.26	1.26	1.26
**C) Cross-sectional desacyl ghrelin**	Intercept	3.60	3.60	3.60	3.60	3.60	3.60
	Std. Error	0.80	0.80	0.80	0.80	0.80	0.80
	Tau.sq	3.55	3.55	3.55	3.55	3.55	3.55
**D) Longitudinal T1T2 total ghrelin**	Intercept	1.71	1.71	1.71	1.71	1.71	1.71
	Std. Error	0.55	0.55	0.55	0.55	0.55	0.55
	Tau.sq	2.69	2.69	2.70	2.70	2.70	2.70
**E) Cross-sectional T2HC total ghrelin**	Intercept	1.58	1.58	1.58	1.58	1.58	1.58
	Std. Error	0.63	0.63	0.63	0.63	0.63	0.63
	Tau.sq	3.46	3.46	3.47	3.47	3.47	3.47
**F) Longitudinal T1T2 acyl ghrelin**	Intercept	-0.24	-0.24	-0.24	-0.24	-0.24	-0.24
	Std. Error	0.28	0.28	0.28	0.28	0.28	0.28
	Tau.sq	0.29	0.29	0.29	0.29	0.29	0.29
**G) Cross-sectional T2HC acyl ghrelin**	Intercept	1.92	1.92	1.92	1.92	1.92	1.92
	Std. Error	1.27	1.27	1.27	1.27	1.27	1.27
	Tau.sq	4.41	4.41	4.41	4.41	4.41	4.41

Results of sensitivity analysis of meta-analysis using robust variance estimation using a default rho of 0.8. Std. Error=Standard error; Tau.sq= Tau squared

Table S9 Meta-regression models including age, assay tape and quality of evidence			
	**Covariate**	**Coefficient**	**p**
**A) Cross-sectional total ghrelin**	Mean Age	-0.05	0.68
	ELISA (EIA reference)	0.63	0.76
	RIA (EIA reference)	1.48	0.48
	Quality	-0.61	0.29
**B) Cross-sectional acyl ghrelin**	Mean Age	-0.29	0.47
	ELISA (EIA reference)	0.11	0.91
	RIA (EIA reference)	-0.65	0.64
	Other (EIA reference)	0.36	0.91
	Quality	0.01	0.99
**C) Cross-sectional desacyl ghrelin**	Mean Age	-0.38	0.44
	ELISA (EIA reference)	10.13	0.24
	RIA (EIA reference)	11.82	0.19
	Quality	-5.40	0.20
**D) Longitudinal T1/T2 total ghrelin**	Age at admission	-0.00	0.99
	RIA (ELISA reference)[a]	-	-
	Quality	-1.04	0.25
**E) Cross-sectional T2/HC total ghrelin**	Mean Age	-0.14	0.69
	RIA (ELISA reference)	2.07	0.44
	Quality	-0.50	0.76
**F) Longitudinal T1/T2 acyl ghrelin**	Age at admission	0.28	0.30
	Other (ELISA reference)	1.23	0.30
	Quality[b]	-0.85	0.26
	Duration to follow-up	-0.01	0.82
**G) Cross-sectional T2/HC acyl ghrelin**	Mean Age	0.00	0.99
	RIA (ELISA reference)	1.62	0.38
	Other (ELISA reference)	6.72	0.13
	Quality[a]	-	-

Results of meta-regression for all calculated meta-analyses. [a]All but one study (ELISA) used RIA for estimating ghrelin values. [b]All but one study had quality level 6.
